# Genomic profiling using the UltraSEEK panel identifies discordancy between paired primary and breast cancer brain metastases and an association with brain metastasis-free survival

**DOI:** 10.1007/s10549-021-06364-8

**Published:** 2021-09-09

**Authors:** Athina Giannoudis, Alexander Sartori, Lee Eastoe, Rasheed Zakaria, Christopher Charlton, Nicholas Hickson, Angela Platt-Higgins, Philip S. Rudland, Darryl Irwin, Michael D. Jenkinson, Carlo Palmieri

**Affiliations:** 1 Institute of Systems, Molecular and Integrative Biology, Molecular and Clinical Cancer Medicine, University of Liverpool, Sherrington Building, Ashton Street, Liverpool, L69 3GE UK; 2 Agena Bioscience GmbH, Hamburg, Germany; 3 Department of Neurosurgery, The Walton Centre NHS Foundation Trust, Liverpool, UK; 4 Manchester University Hospital NHS Foundation Trust, Manchester, UK; 5 Institute of Systems, Molecular and Integrative Biology, Pharmacology and Therapeutics, University of Liverpool, Liverpool, UK; 6 The Clatterbridge Cancer Centre NHS Foundation Trust, Liverpool, UK

**Keywords:** UltraSEEK®, Mutations, Breast cancer, Brain metastasis

## Abstract

**Purpose:**

Brain metastases (BM) are an increasing clinical problem. This study aimed to assess paired primary breast cancers (BC) and BM for aberrations within TP53, PIK3CA, ESR1, ERBB2 and AKT utilising the MassARRAY® UltraSEEK® technology (Agena Bioscience, San Diego, USA).

**Methods:**

DNA isolated from 32 paired primary BCs and BMs was screened using the custom UltraSEEK® Breast Cancer Panel. Data acquisition and analysis was performed by the Agena Bioscience Typer software v4.0.26.74.

**Results:**

Mutations were identified in 91% primary BCs and 88% BM cases. TP53, AKT1, ESR1, PIK3CA and ERBB2 genes were mutated in 68.8%, 37.5%, 31.3%, 28.1% and 3.1% respectively of primary BCs and in 59.4%, 37.5%, 28.1%, 28.1% and 3.1% respectively of BMs. Differences in the mutations within the 5 genes between BC and paired BM were identified in 62.5% of paired cases. In primary BCs, ER-positive/HER2-negative cases harboured the most mutations (70%), followed by ER-positive/HER2-positive (15%) and triple-negatives (13.4%), whereas in BMs, the highest number of mutations was observed in triple-negative (52.5%), followed by ER-positive/HER2-negative (35.6%) and ER-negative/HER2-positive (12%). There was a significant association between the number of mutations in the primary BC and breast-to-brain metastasis-free survival (*p* = 0.0001) but not with overall survival (*p* = 0.056).

**Conclusion:**

These data demonstrate the discordancy between primary BC and BM, as well as the presence of clinically important, actionable mutations in BCBM. The UltraSEEK® Breast Cancer Panel provides a tool for BCBM that can be utilised to direct more tailored treatment decisions and for clinical studies investigating targeted agents.

**Supplementary Information:**

The online version contains supplementary material available at 10.1007/s10549-021-06364-8.

## Introduction

Breast cancer brain metastasis (BCBM) is a growing clinical problem associated with significant morbidity and mortality [1]. Up to 40–50% of women with HER2-positive and triple-negative breast cancers (BC) will develop brain metastasis (BM), whereas its incidence in hormone receptor-positive BC is 14% [2–5]. The application of next generation sequencing technologies has enabled the characterisation of BCs and highlighted that BC subtypes differ in their mutational profiles but overall, the commonest mutated genes are TP53, PIK3CA, GATA3, CDH1, AKT1 [6–11]. Genomic profiling has also demonstrated the complex and diverse molecular landscapes of secondary metastatic BCs [7, 8, 11, 12]. BM sequencing studies have identified differences in their mutational landscape as compared to primary tumours [13–17]. The identification of genomic alterations within BM and the use of targeted therapies against these mutations may improve the clinical outcomes of patients with BCBM [10–17].

In the era of precision medicine and targeted therapies, the choice of anti-cancer therapy is increasingly tailored according to the molecular and/or genomic characterisation of the underlying malignancy. Identifying rare and low-level mutations due to tumour heterogeneity in cancer samples and/or of poor-quality DNA isolated from formalin-fixed, paraffin-embedded (FFPE) tissues entails tools that offer both low-frequency detection and efficient use of starting material. Such tools could, in turn, be utilised for therapeutic decision making and/or development of clinical trials using targeted agents. The UltraSEEK® (Agena Bioscience, San Diego, USA) technology provides a targeted, multiplexed method for detecting rare events, with detection threshold as low as 0.1% on the MassARRAY® system utilising DNA isolated from FFPE tissues, plasma and cerebrospinal fluid [18–21]. Moreover, multiplex detection of low-frequency mutations is becoming a necessary diagnostic tool for clinical laboratories that must overcome several challenges such as the detection of minor alleles among abundant wild-types, the heterogeneous nature of the tumours and the limited amount of available tissue [18–21]. The UltraSEEK® Breast Cancer panel has been developed to enable the assessment of five commonly mutated genes in breast cancer, TP53, PIK3CA, ERBB2, ESR1 and AKT1. This study aimed to assess the mutational landscape of paired BC and BMs for these known key genomic drivers, utilising the UltraSEEK® BC panel.

## Materials and methods

### Patients

A total of 32 FFPE primary BC samples, with their paired BMs were collected from the Liverpool Tissue Bank and the Walton Research Tissue Bank (WRTB) Liverpool, UK. The cases were stained by immunohistochemistry (IHC) for hormone receptor expression (ER, PgR) and HER2 as previously described [22]. The study was performed in accordance with the Declaration of Helsinki and approved by the WRTB (WRTB 15_06) and the National Research Ethics Committee (NRES 11/WN003/2). Written consent was in place before anonymised tissue and data were released for research purposes [22].

### DNA extraction and mutation profiling

DNA was isolated from 32 BC and their matched BM cases using the GeneRead DNA FFPE kit (Qiagen, Crawley, UK) and screened using the custom UltraSEEK BC Panel (Agena Bioscience, San Diego, USA). Starting from a single global multiplex polymerase chain reaction (PCR) the panel tests 39 mutations (Table 1) across 5 oncogenes (TP53, PIK3CA, ESR1, ERBB2 and AKT) in 8 multiplex assays (Supplementary table 1). The number of mutations was defined as the total number of mutations identified in any of the 5 genes per patient. The range of mutations is 0–39 (Table 1). PCR was performed using 10 ng of DNA according to the manufacturer’s instructions (Agena Bioscience, San Diego, USA). Amplified products were treated with shrimp alkaline phosphatase (SAP) and the PCR/SAP product was aliquoted in a new 96-well plate for downstream extension and termination reaction according to the manufacturer’s instructions. The single-base extended oligonucleotides were captured by streptavidin-coated magnetic beads and biotin-labelled following manufacturer’s instructions. The products were then transferred to the MassARRAY System with Chip Prep Module 96 (CPM96) that automatically performs desalting (resin), transfer of analyte and calibrant to the SpectroCHIP® Arrays and loading of SpectroCHIP® Arrays. Data were automatically acquired using the MassARRAY Analyzer. The workflow is presented and summarised in Supplementary Fig. 1.

**Table 1:** UltraSEEK breast cancer panel

Genes	Missense mutations	No of mutations
AKT1	pE17K٭, pL52R٭	2
ERBB2 (HER2)	pG309A, pG309E, pS310F٭, pL755R, pL755S, pL755_T759del , pD769H٭, pD769Y, pV777L٭, pL869R٭	10
ESR1	pA283V, pK303R, pE380Q٭, pV392I, pS463P, pL536R, pL536Q, pY537C, pY537N٭, pY537S, pD538G, pS576L	12
PIK3CA	pN345K, pC420R٭, pE542K٭, pE545A, pE545K٭, pE545Q, pH1047L٭, pH1047R	8
TP53	pR175H, pR213X, pY220C, pR248Q, pR248W٭, pR273C, pR273H	7
Total	5 genes	39

The UltraSEEK BC panel screens for 39 mutations across 5 common BC oncogenes

*Multiple assays for these mutations are included in the panel

**Table 2:** OncoKB database and gene actionability

Tumour type: breast cancer						
Level	Alteration: oncogenic mutations	No of mutations (%)		Drugs	Citations	
		BC (*N* = 32)	BM (*N* = 32)			
1	PIK3CA	10 (15)	10 (17)	Alpelisib + Fulvestrant	3	PI3K inhibition results in enhanced oestrogen receptor function and dependence in hormone receptor-positive breast cancer. Bosch A et al. Sci Transl Med. 2015 PMID: 25,877,889
						Alpelisib Plus Fulvestrant in PIK3CA-Altered and PIK3CA-Wild-Type Oestrogen Receptor-Positive Advanced Breast Cancer: A Phase 1b Clinical Trial. Juric D et al. JAMA Oncol. 2019 PMID: 30,543,347
						Alpelisib for PIK3CA -Mutated, Hormone Receptor-Positive Advanced Breast Cancer. André F et al. N Engl J Med. 2019 PMID: 31,091,374
3				GDC-0077	3	355TiP Phase III study of GDC-0077 or placebo (pbo) with palbociclib (P) + fulvestrant (F) in patients (pts) with PIK3CA-mutant/hormone receptor-positive/HER2-negative locally advanced or metastatic breast cancer (HR + /HER2– LA/MBC). TurnerN et al. Annals Onc. 2020 10.1016/j.annonc.2020.08.457
						Juric D et al. Abstract# OT1-08–04, SABCS 2019
						Hong R et al. Abstract# PD4-14, SABCS 2017
3				Copanlisib + Fulvestrant	7	First-in-human phase I study of copanlisib (BAY 80–6946), an intravenous pan-class I phosphatidylinositol 3-kinase inhibitor, in patients with advanced solid tumours and non-Hodgkin's lymphomas. Patnaik A et al. Ann Oncol. 2016 PMID: 27,672,108
						Exceptional Response to Copanlisib in a Heavily Pretreated Patient With PIK3CA-Mutated Metastatic Breast Cancer. Spathas N et al. JCO Prec Onc. 2020 10.1200/PO.19.00049
						Staben et al. Abstract# DDT02-01, AACR 2017
						De et al. Abstract# 3438, AACR 2019
						O'Brien, NA et al. Abstract P3-04–15. Cancer Research, 2017
						De et al. Abstract# P2-03–08, SABCS 2018
						Edgar et al. Abstract# 156, AACR 2017
3	AKT1	12 (18)	12 (20.3)	AZD5363	4	Preclinical pharmacology of AZD5363, an inhibitor of AKT: pharmacodynamics, antitumour activity, and correlation of monotherapy activity with genetic background. Davies BR et al. Mol Cancer Ther. 2012 PMID: 22,294,718
						Discovery of 4-amino-N-[(1S)-1-(4-chlorophenyl)-3-hydroxypropyl]-1-(7H-pyrrolo[2,3-d]pyrimidin-4-yl)piperidine-4-carboxamide (AZD5363), an orally bioavailable, potent inhibitor of Akt kinases. Addie M et al. J Med Chem. 2013 PMID: 23,394,218
						AKT Inhibition in Solid Tumours With AKT1 Mutations. Hyman DM et al. J Clin Oncol. 2017 PMID: 28,489,509
						Tumours with AKT1E17K Mutations Are Rational Targets for Single Agent or Combination Therapy with AKT Inhibitors. Davies BR et al. Mol Cancer Ther. 2015PMID: 26,351,323
1	ERBB2/HER2	1 (1.5)	1 (1.7)	Neratinib	3	Activating HER2 mutations in HER2 gene amplification negative breast cancer. Bose R et al. Cancer Discov. 2013 PMID: 23,220,880
						The major lung cancer-derived mutants of ERBB2 are oncogenic and are associated with sensitivity to the irreversible EGFR/ERBB2 inhibitor HKI-272. Minami Y et al. Oncogene. 2007 PMID: 17,311,002
						HER kinase inhibition in patients with HER2- and HER3-mutant cancers. Hyman DM et al. Nature. 2018 PMID: 29,420,467
3	ESR1	13 (19.4)	11 (18.6)	AZD9496	2	Efficacy of a novel orally active SERD AZD9496 against hormone dependent post-menopausal breast cancer depends on inhibition of cellular aromatase activity. Kazi A et al. J Ster Biochem Mol Biol. 2020 ISSN: 0960–0760
						A Randomised, Open-label, Presurgical, Window-of-Opportunity Study Comparing the Pharmacodynamic Effects of the Novel Oral SERD AZD9496 with Fulvestrant in patients with Newly Diagnosed ER + HER2 − Primary Breast Cancer. Robertson JFR et al. 2020 PMID: 32,234,755
1				Abemaciclib + Fulvestrant	4	Analysis of Overall Survival Benefit of Abemaciclib Plus Fulvestrant in Hormone Receptor–Positive, ERBB2-Negative Breast Cancer. Gil-Sierra MD et al. JAMA Oncol. 2020 10.1001/jamaoncol.2020.1516
						The Effect of Abemaciclib Plus Fulvestrant on Overall Survival in Hormone Receptor-Positive, ERBB2-Negative Breast Cancer That Progressed on Endocrine Therapy-MONARCH 2: A Randomised Clinical Trial. Sledge GW Jr et al. JAMA Oncol. 2019 PMID: 31,563,959
						Activating ESR1 Mutations Differentially Affect the Efficacy of ER Antagonists. Toy W et al. Cancer Discov. 2017 PMID: 27,986,707
						Plasma ESR1 Mutations and the Treatment of Oestrogen Receptor-Positive Advanced Breast Cancer. Fribbens C et al. J Clin Oncol. 2016 PMID: 27,269,946
NA	TP53	31 (46.3)	25 (42.4)			
Total no of mutations		67	59			

A search on the oncoKB for the actionability of PIK3CA, AKT1, ESR1, ERBB2/HER2 and TP53 oncogenic mutations on breast cancer identified 7 protocols. 1. FDA-approval, 3. Clinical evidence

*NA* Not available

### Data analysis and statistics

Data were analysed using the Typer software v4.0.26.74 (Agena Bioscience, San Diego, USA). The signal intensity of the mutant allele was normalised against the capture-control peaks (biotin-labelled, non-reactive oligos, used as an internal control) as previously reported [18, 19]. Samples with a mutant allele-call with a signal-to-noise ratio ≥ 6 and a z-score ≥ 7 were considered positive for the mutation. Positive mutant-calls with z-scores 7–10 were labelled ‘L’ (low-level confidence) to distinguish from the results with high-level confidence (T), z-score > 10 [18–20]. The z-score is a robust scoring representing the deviation of the minor allele’s frequency from the median baseline frequency, as measured by median absolute deviation (MAD) units. The baseline signal distribution or population of that peak is generated by analysing a population of known wild-type samples for each target and capture pre-analytical and background noise inherent to the assay and analyte. The UltraSEEK chemistry and methodology has been analytically presented by Mosko et al. [19].

RAWGraphs (https://rawgraphs.io/) was used to generate the alluvial diagram illustrating the receptor switching between the primary and metastatic setting. Fisher’s Exact test was used to compare the receptor status change versus the number of mutations (≤ 3 vs > 3) in the primary BC and Wilcoxon signed-rank paired *t* test (Gaussian approximation) was used to compare the total number of mutations between the paired BC and BMs. Kaplan–Meier (Log-rank) survival analysis was used to determine whether the number of mutations in the primary BC and the receptor status change between BCs and BMs was associated with the breast–brain metastasis-free survival (BMFS, time between the initial breast surgery and the resection of the BM) and overall survival (OS, time between breast diagnosis/surgery and death from any cause). Given the range of mutations seen in primary breast cancer samples was 0–5 and to enable the greatest contrast within the studied population an arbitrary cut-off of > 3 mutations was chosen following an analysis of groups based on 0–1, 2–3 and 4–5 (> 3) mutations in the primary breast cancer (Supplementary Fig. 2). Statistical analysis was performed on GraphPad Prism v5.0 (GraphPad Inc, San Diego, USA).

The OncoKB (http://oncoKB.org) and ClinicalTrials.gov (https://clinicaltrials.gov) databases were used to identify actionability and ongoing clinical trials on BC using targeted therapies for the mutated genes in this study. The searches on OncoKB were performed for Breast Cancer, Actionable Genes and the following Levels of Evidence: 1. FDA-approval, 2. Standard care, 3. Clinical evidence, 4. Biological evidence, R1/R2. Resistance. The searches on the ClinicalTrials.gov were performed for Breast Cancer, Recruiting, Active (not recruiting), Completed, Adult and selecting for specific gene mutations/alterations for PIK3CA, AKT1, ESR1, ERBB2/HER2 and TP53. Similar work on BCBM mutated genes was recently presented by our group in a systematic review [14].

## Results

### Patient characteristics and receptor switching

**Fig. 1:**
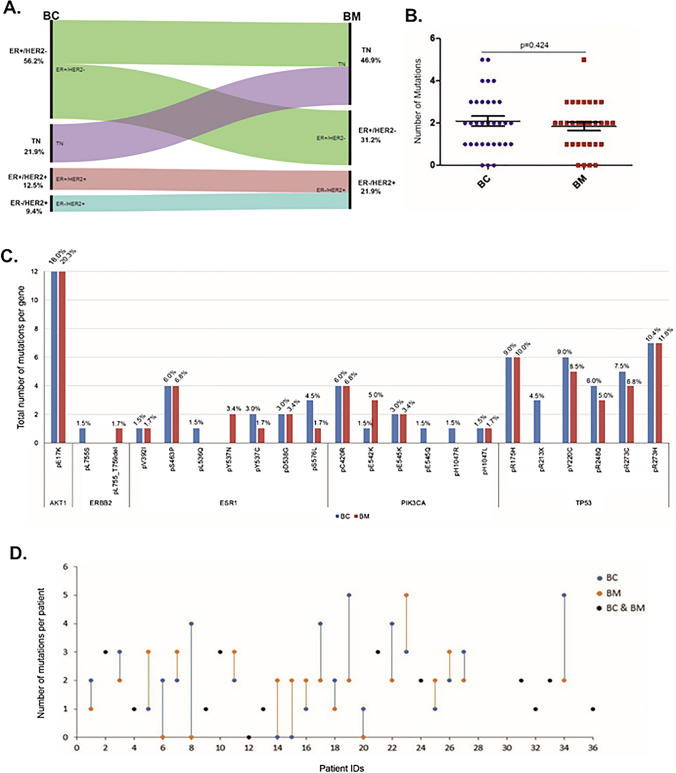
Receptor switch and mutation detection in paired BC and BM samples. **A** The Alluvial diagram illustrates the receptor (ER, PgR, HER2) switch between primary BC and their paired BM cases including % for each subtype. **B** No significant association was identified (*p* = 0.484) in the number of mutations between primary BCs and their paired BMs. **C** Total number of mutations per gene identified in both BC (blue) and BM (red) samples. The percentage of each mutation over the total number of mutations is presented on the top of each bar and on supplementary table 3. **D** Scatter-plot showing the number of mutations for each pair. Blue dots-lines and red dots-lines represent the number of mutations changing between BC and from BM. Black dots represent the cases with similar number of mutations in both the primary BC and its paired BM

Among the 32 primary BCs, 18 (56.2%) were ER-positive/HER2-negative, 4 (12.5%) were ER-positive/HER2-positive, 3 (9.4%) were ER-negative/HER2-positive and 7 (21.9%) were triple-negative (TN). Eight of the 18 (44.4%) ER-positive and all the 4 (100%) ER-positive/HER2-positive primary BCs lost ER expression in BM. The HER2-positive and TN primary BCs maintained their receptor status in the BM. The changes of the receptor status between BCs and their paired BMs are illustrated in the alluvial diagram (Fig. 1A).

### Mutational profiling of primary BCs and their paired BMs

A mutation in any of the five genes was identified in 29 of 32 (90.6%) primary BCs and 28 of 32 (87.5%) BM cases. A total number of 67 and 59 mutations were detected in primary BCs and their paired BMs respectively without significant association in the number of mutations between the paired BC and BM cases (*p* = 0.424, median 2 in both BC and BM, Fig. 1B). Out of the 32 paired BCs and BMs, TP53, AKT1, ESR1 and PIK3CA genes were respectively mutated in 22 (68.8%), 12 (37.5%), 10 (31.3%) and 9 (28.1%) of the primary BCs and in 19 (59.4%), 12 (37.5%), 9 (28.1%) and 9 (28.1%) of the BMs. ERBB2 mutations were detected in 1 out of 32 (3.1%) BC and BM cases. The total number of mutations in each gene, including percentages, is illustrated in Fig. 1C and Supplementary table 2.

### Differences in the number of mutations between primary BCs and their paired BMs

**Fig. 2:**
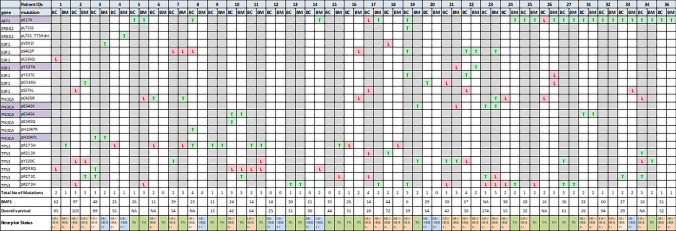
The genomic landscape of BCBM using the UltraSEEK Breast Cancer Panel. The illustrated matrix presents paired BC and BM patients IDs, the genes with the identified mutations, the multiplex assays (purple indicates that 2 assays were run for these mutations) and the confidence call of the mutations. T (green): High level confidence call = high signal intensity and z-score > 10, L (red): Low level confidence call = low signal intensity and z-score 7–10. The total number of mutations, the breast–brain metastasis-free (BMFS) and the overall survival (both in months) and the receptor status are also presented. * Synchronous, *NA* Not available

**Fig. 3:**
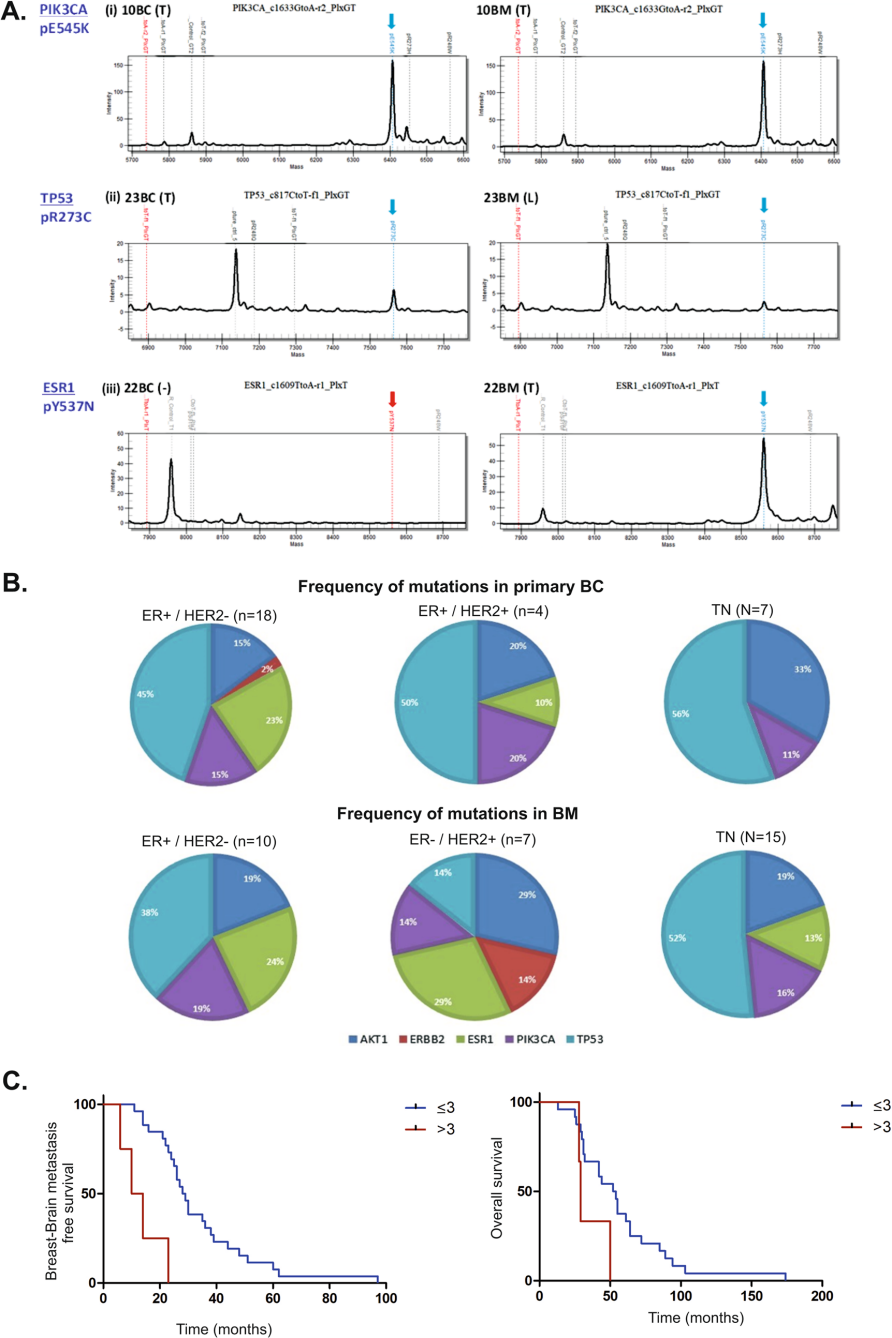
Mutation detection in paired BC and BM samples. **A** Representative examples of mutation spectra between paired BC/BM samples with identified mutations as (i) T/T, (ii) T/L and (iii) (-/T) with relative intensity on the y-axis and mass/charge on the x-axis. **B** The frequency (percentages) of the identified mutations for each of the 5 genes according to the ER, PgR and HER2 receptor status in primary BC and in paired BM. The exact number of mutations is presented in Supplementary Table 4. One of the 3 ER-negative/HER2-positive primary BC samples was carrying an ESR1 mutation, whereas no mutations were identified in the other 2 samples and they were not included in the pie-charts. **C** Kaplan–Meier (Log-rank) survival plots of breast–brain metastasis-free survival (BMFS) and overall survival (OS). Patients carrying ≤ 3 mutations had a significantly better BMFS (*p* = 0.0001, HR: 0.011, 95% CI: 0.001–0.112), than patients with > 3 mutations. There was no association with OS (*p* = 0.056, HR: 0.146, 95% CI: 0.020–1.054) and number of mutations

We observed that in 20 of 32 (62.5%) paired cases there were differences in the total number of mutations between the primary BC and paired BM with 11 of 32 (34.4%) and 9 of 32 (28.1%) cases showing either a reduction or increase in the total number of mutations respectively in the paired BMs. In 12 of 32 (37.5%) paired cases the number of mutations did not differ. The scatter-plot showing the number of mutation changes between pairs is illustrated in Fig. 1D. The genomic landscape of the paired BC and their BMs for the individual mutations and the confidence level of detection (T or L) for each one is presented in a matrix format in Fig. 2. We observed that 22/32 (68.75%) of patients lost or gained a mutation in the brain metastatic site in at least one of the 5 clinically relevant genes (Fig. 2). Mutations were detected with similar confidence (T/T and/or L/L) in 18 of 32 (56.3%) paired BC and BM cases and increased to 21 of 32 (65.6%) when mutations with different confidence (L/T) between paired BC and BM cases were included, highlighting the differences in the genomic landscape between primary BC and paired BM. For instance, the AKT1 pE17K mutation was detected in 10 of 32 (31.25%) paired cases; 8 of 32 (25%) with the same confidence levels and 2 of 32 (6.3%) cases with low confidence (L) in the primary and high confidence (T) in paired BM (Fig. 2). The TP53 pR273H was the more prevalent mutation in both BC and BM cases present in 4 of 32 (12.5%) paired cases, 3 of which with similar confidence and 1 with lower confidence in BC than BM (Fig. 2). Representative examples of the different mutation spectra highlighting the differences in the confidence levels (T/T, T/L, -/T) are presented in Fig. 3A.

### Mutations and receptor status change between primary BCs and their paired BMs

The 32 primary BCs contained a total of 67 mutations and hormone receptor-positive cases harboured the most mutations (47/67; 70%), followed by ER-positive/HER2-positive (10/67; 15%) and triple-negatives (9/67; 13.4%), whereas in BMs with a total of 59 mutations, the highest number was observed in triple-negatives (31/59; 52.5%), followed by hormone receptor-positive (21/59; 35.6%) and HER2-positive (7/59; 12%). Of the 32 primary BCs, 27 (84.37%) had 3 or less mutations (≤ 3) and 9 of these (33.3%) change status in BM, whereas 18 (66.7%) maintain their receptors. Of the 5 (15.63%) primary BCs with > 3 mutations, 3 (60%) change status in BM and 2 (40%) maintain their receptors. There was no significant association between the receptor switching and the number of mutations identified by two-tail Fisher’s exact test (*p* = 0.337). The number of patients, the total number of mutations and the number of mutations in each gene according to the receptor status of BC and BM are presented in Supplementary table 2. All the mutation frequencies for the primary BCs and BMs according to the receptor status are illustrated in Fig. 3B.

### Association of mutations with patient outcome

Patients with primary BCs with ≤ 3 mutations had a significantly longer BMFS, than patients whose BCs had > 3 mutations with median BMFS of 28.5 and 12 months respectively (*p* = 0.0001, HR: 0.011, 95% CI: 0.001–0.112, Fig. 3C). There was no association with OS although the median OS was 53 and 29 months in patients carrying ≤ 3 and > 3 mutations respectively (*p* = 0.056, HR: 0.146, 95% CI: 0.020–1.054, Fig. 3C). Patients with differences in the numbers of mutations between primary BC and BM classified as mutation numbers increasing in BM versus mutation numbers decreasing in BM, showed a significant difference in the brain metastasis-death (time between brain metastasis and death due to any cause) survival outcome (*p* = 0.0008, HR: 17.72, 95% CI: 3.277–95.87, Supplementary Fig. 3).

When the patients were classified according to the change in ER, PgR and HER2 status between primary BC and BM the median BMFS was 24.5 (receptor change) and 29.5 (no change) (*p* = 0.115) months. No significant association was observed with OS (*p* = 0.618) either.

### Potentially actionable targets

A search on oncoKB for the actionability of PIK3CA, AKT1, ESR1, and ERBB2/HER2 oncogenic mutations on BC identified 7 protocols, 3 of which are FDA-approved (Alpelisib, Neratinib and Abemaciclib. No protocols were identified for TP53 (Table 2).

Forty-three clinical trials are currently available for BC patients with mutations in these 5 genes as identified within ClinicalTrials.gov (Supplementary table 3), whereas twelve trials specifically recruiting patients with BCBM using targeted therapies for the identified gene mutations/alterations were recently reported [14]. Drugs targeting PIK3CA [Paxalisib (GDC-0084), Buparlisib (BMK120)], ERBB2/HER2 (Neratinib, Afatinib, Pyrotinib, Tucatinib), pathways and/or genes involved in the cell cycle (Abemaciclib, NKTR-102) are currently undergoing assessment in clinical trials either as single agents or in combination with standard of care treatments for BCBM [14].

## Discussion

In this study, we assessed for the presence of mutations within key BC genes utilising the UltraSEEK® technology in a paired cohort of BCs and BMs. A mutation in at least one of the five genes (TP53, PIK3CA, ERBB2, ESR1, AKT1) was identified in most cases, with 90.6% of primary BC and 87.5% of the paired BMs having at least one mutation. The observed similarities regarding the frequency of the mutated genes reflect the nature of the UltraSEEK® BC panel that was designed to target actionable genes present in all the key BC subtypes. Twenty-two out of the 32 patients in this study (68.75%), lost or gained a mutation in the brain metastatic site in at least one of the 5 clinically relevant genes (Fig. 2) highlighting the importance of screening for mutations in the primary and metastatic site. The actionability of these mutated genes in primary BC and BM is presented in Table 2 and in a recent systematic review [14]. The detection of ESR1 mutations in the primary tumours also highlights the sensitivity of the assay [19]. Although ESR1 mutations were more commonly identified in metastatic BCs [23, 24], the use of the sensitive droplet-digital PCR reported higher frequencies in primary tumours [25, 26]. The identification of therapeutic strategies in breast cancers harbouring ESR1 mutants is an area of active interest. Fulvestrant has demonstrated poor clinical activity in ESR1-mutated BC [27, 28], whereas bazedoxifene and lasofoxifene have demonstrated activity in pre-clinical models of ESR1-mutated BC [29, 30]. The efficacy of lasofoxifene is currently being explored in the ELAINE trial (NCT03781063) in patients with ESR1-mutated BCs (Supplementary table 3). Interestingly, in our cohort, ESR1 mutations were seen in 28.1% of BMs despite the loss of ER expression by IHC, indicating that the ESR1-mutant clones are likely dominant clones, resistant to therapy [23–26, 28]. These results are in accordance with others who detected a high ESR1 mutation frequency (34.3–44.9%) in BMs [25].

While TP53 is mutated in all BC subtypes, it is most common in TNs and HER2 [4, 31–33]. TP53 mutations have been associated with worse clinical outcomes and poor response to hormonal therapy, chemotherapy and/or radiotherapy [31–34] and our samples have been acquired from patients who had progressed and developed brain metastasis despite prior treatment. TP53 can be re-activated by targeting molecules that modulate its posttranslational modifications, localisation and degradation and several ongoing clinical trials are using TP53-reactivating compounds in combination with chemotherapeutic drugs (Supplementary table 3) [35, 36].

ERBB2/HER2 mutations were identified in only 2 samples, a primary ER-positive BC and a HER2-positive BM. These ERBB2/HER2 mutations are associated with resistance to lapatinib but are sensitive to neratinib, highlighting the importance of treating HER-mutated cancers with the appropriate HER-targeted drugs (Table 2, Supplementary table 3) [37, 38]. The identification of mutations in PIK3CA and AKT1 are also of clinical significance since both genes are druggable (Table 2, Supplementary table 3). The PI3Kα-specific inhibitor alpelisib, has shown activity in PIK3CA-mutant breast cancers (NCT02437318) and recently granted FDA and European commission approval, while its potential in the regression and stabilisation of progressive BCBM has been highlighted [39, 40]. The brain-penetrant inhibitor paxalisib (GDC-0084) has demonstrated activity in pre-clinical models of BCBM [41]. Several inhibitors targeting the AKT1 pE17K mutation, an oncogenic driver in BC, have shown efficacy as monotherapy or in combination with other drugs (Table 2, Supplementary table 3) [42–44]. We recently presented a summary of current clinical trials on mutated BCBMs [14].

Several studies have identified large similarities in the mutational profiles of primary tumours and their metastases including BMs [6–11], whereas others showed clear differences between primary and metastasis in the numbers and types of mutations [12–15]. It was recently suggested that the systemic metastatic seeding can begin early during primary tumour growth and that the clonal architecture is remodelled by treatment that may select for disseminated cells harbouring resistant mutations [45]. Treatment was also associated with high gene heterogeneity and monoclonal metastases [45]. Similarly, within our cohort of paired BC and BM cases, the median BMFS time (irrespectively of mutation status) was 26 months and certain gene mutations were detected with similar confidence levels in 56.3% of paired cases (monoclonal metastases). We also identified cases where the mutation was absent or present with low confidence in the primary and with high confidence in the BM and vice versa. This is clinically relevant as the identification of mutations especially with low intensity (low confidence levels) in the primary could indicate the presence of clones that could drive the metastatic process or be responsible for treatment-resistance and present therefore with higher intensity (high confidence levels) in the metastatic site. Targeting these mutations early, in the primary disease with the appropriate drugs could possibly prevent the development of metastatic disease. Similarly, the absence of mutations in the primary tumour that are present in the metastatic site (low or high) is of clinical relevance as these mutations should be targeted with the appropriate therapeutic regimens aimed at the brain metastatic site. The fact that only 37.5% of mutations in the 5 chosen genes are conserved between the primary and just one major metastatic site, the brain, suggests that, in general, compounds targeting these mutations identified solely in the primary breast tumour are unlikely to be successful in the majority of advanced/metastatic patients. Therefore, it seems to be necessary to sample metastases, as well as the primary tumour to identify somatic mutations to tumours at both sites, to predict more accurately whether a patient will respond to such chemical interventions. The differences in the mutational landscape could be attributed to the clonal evolution process, the selective pressure of different therapeutic regimens and the receptor switching between primary BC and metastasis. Evidence of clonal remodelling between primary tumours and metastases associated with the clinical subtype conversion was recently presented, but the most frequently mutated genes in primary tumours were also identified in metastases independent of the tumour subtype [12]. We did not observe significant differences in the frequently mutated genes in relation to the subtypes in BC and BM and there was no significant association between receptor switching and number of mutations. This could also be attributed to the small number of genes present in the targeted BC mutation panel and the small number of mutations identified in our cohort. Nevertheless, within our cohort, patients carrying ≤ 3 mutations had a significantly better BMFS (*p* = 0.0001), than patients with > 3 mutations indicating that the higher number of mutations correlates to worst prognosis (metastasis-free survival).

A limitation of the MassARRAY UltraSEEK technology and therefore, a limitation of this study, in comparison to next generation sequencing on the identification of mutations is the use of predefined mutations across different oncogenes. Although, it cannot detect unknown mutations and copy number alterations, it is more cost-effective and could be easier applied in a clinical setting [21, 46, 47]. Another limitation is the number of paired samples in the study, although it is higher that the number of paired BCBM samples reported in other genomic studies [14]. The challenges to obtain BM samples for sequencing including the inherent risks of neurosurgery, as samples can only be taken when surgical resection is clinically indicated, make longitudinal studies of the changes in the BM genome unethical. The increasing use of stereotactic surgery (SRS) has reduced the need for surgical resection but also, the availability of tissue. The alternative use of circulating cell-free DNA (cfDNA) in the CSF and plasma is under investigation and in the future patients will be able to have tailored treatments based on the results of sequencing cfDNA from CSF rather than relying on the genomics of the primary lesion [20, 46, 47]. Nevertheless, these data complement the current literature as it highlights the presence of actionable mutations in clinically important genes both in BC and BM identified by a sensitive, targeted, comprehensible technology, potentially useful in a routine clinic. The fact that the limited number of mutations detected and the change in the number of mutations in the metastatic site correlate to survival outcomes reinforces the importance of patient mutation screening.

In summary, our data highlight the presence of clinically important and actionable mutations in AKT1, ESR1, PIK3CA, TP53 and ERBB2 genes in BC and in BCBM as identified by the UltraSEEK BC panel that provides a powerful tool to investigate low abundance mutations and could be potentially useful in a clinical environment [25, 26]. These mutations could be used to identify patients resistant to certain therapeutic regimens and enable the development of more tailored clinical studies utilising targeted agents or combinations of them in the brain metastatic setting.

## Supplementary Information

Below is the link to the electronic supplementary material.

Supplementary file1 (DOCX 672 kb)

## Data Availability

All data generated or analysed during this study are included in this published article and its supplementary information files.

## Abbreviations

BC: Breast cancer
BCBM: Breast cancer brain metastasis
BM: Brain metastasis
BMFS: Breast–brain metastasis-free survival
CPM96: Chip prep module 96
ER: Oestrogen receptor
FFPE: Formalin-fixed, paraffin-embedded
HER2: Human epidermal growth factor receptor 2
IHC: Immunohistochemistry
OS: Overall survival
PCR: Polymerase chain reaction
PgR: Progesterone receptor
SAP: Shrimp alkaline phosphatase
TN: Triple-negative
UNG: Uracil-DNA glycosylase
