# Supplementary material for: Genomic profiling using the UltraSEEK panel identifies discordancy between paired primary and breast cancer brain metastases and an association with brain metastasis-free survival
Source: Breast Cancer Res Treat. 2021 Sep 9;190(2):241–53. doi: 10.1007/s10549-021-06364-8 (PMC8558178; doi:10.1007/s10549-021-06364-8)
Supplement: Supplementary file 1 — Supplementary file1 (DOCX 672 kb) [file 10549_2021_6364_MOESM1_ESM.docx]

**Supplementary Figures and Tables**

**Supplementary Figure 1:**

**
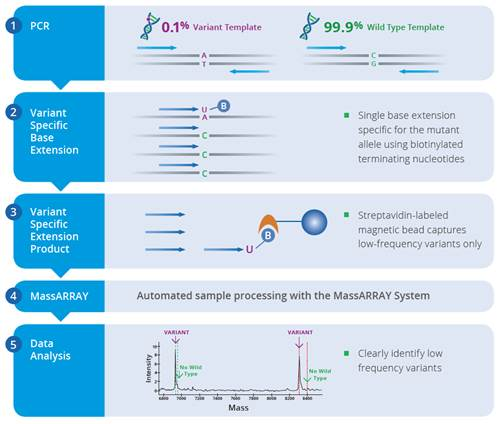
**

**Supplementary Figure 1. Workflow of the UltraSEEK mutation-detection assay.** The UltraSEEK assay consists of multiplex PCR reaction (1) followed by variant-specific extension reaction (2) using biotinylated chain terminator nucleotides and streptavidin-coated magnetic beads (3) that capture the variants. The products are transferred to the automated MassARRAY System (4) that performs desalting and loading of SpectroCHIP Arrays. Data is acquired via matrix-assisted laser desorption/ionization time-of-flight mass spectrometry using the MassARRAY Analyzer (5).

**Supplementary Figure 2:**

**
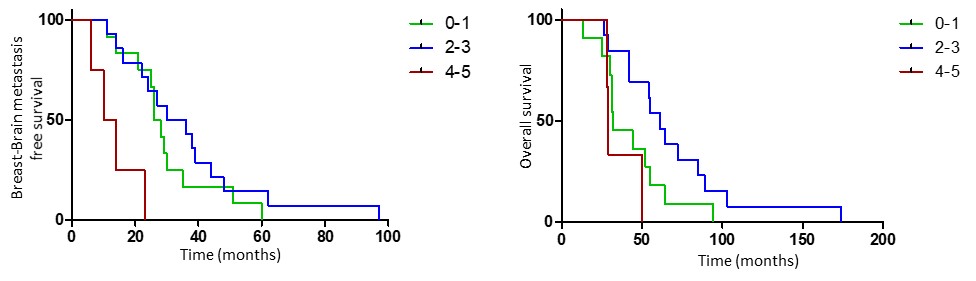
**

**Supplementary Figure 2. Mutations and survival.** Kaplan-Meier survival plots of breast-brain metastasis-free survival (BMFS) and overall survival (OS) showed that patients carrying 0-1 and 2-3 mutations had a significantly better BMFS than patients with 4-5 (>3) mutations (p=0.001, df 2, x^2^=13.86). There was no association with OS (p=0.0517, df 2, x^2^=5.923) and number of mutations. The plots indicate percentage of patients with primary breast cancer (y-axis) and their different survival times (x-axis) to BM diagnosis/surgery and to death.

**Supplementary Figure 3:**

**
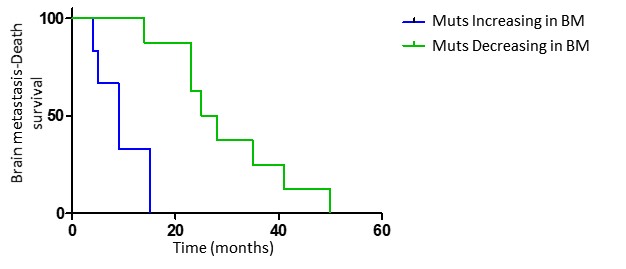
**
**Supplementary Figure 3. Kaplan-Meier survival plot of Brain metastasis to Death survival.** In paired cases where there was a decrease in the number of mutations from the primary BC to BM the survival outcome is better than in paired cases where there was an increase in the number of mutations in BM (p=0.0008, HR:17.72, 95%CI: 3.277-95.87). The plot indicates percentage of patients with brain metastasis (y-axis) and their different survival times (x-axis) from BM diagnosis/surgery to death.

**Supplementary table 1: Multiplex assays of the** **UltraSEEK® Breast Cancer Panel.** The 39 mutations across 5 oncogenes present in the UltraSEEK BC are analysed in 8-plexes (W1-W8) in a 96-well plate format. Fifty-four assays are listed in the table as several gene mutations are interrogated by more than one assay.


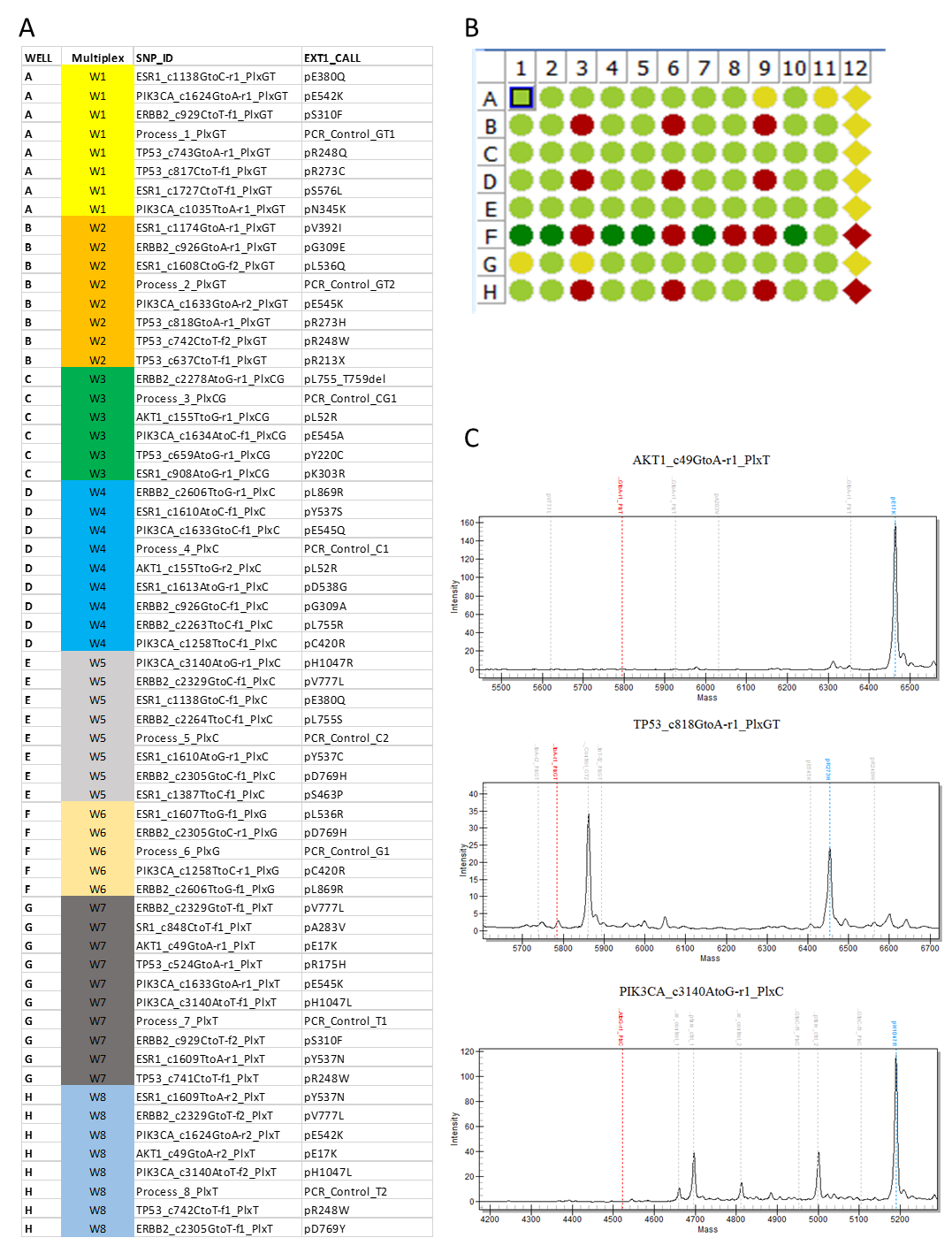


**Supplementary table 2. Mutations in paired BC and BM samples according to receptor status.** The number of mutations identified for each of the 5 genes according to receptor status in **(A)** primary breast cancer (BC) and in **(B)** paired brain metastases (BM). The percentages of each mutation over the total number of mutations is presented in the parenthesis.

**Supplementary table 3.** **List of BC Clinical Trials using targeted therapies.** The searches were performed for Breast Cancer, applying the following filters: Recruiting, Active (not recruiting), Completed, Adult and selecting for specific gene mutations/alterations for PIK3CA, AKT1, ESR1, ERBB2/HER2 and TP53.

| **Altered Gene /Pathway** | **Clinical Trial** | **ClinicalTrials.gov Identifier** | **Phase** | **Targeted Therapy** |
| --- | --- | --- | --- | --- |
| **PIK3CA** | Phosphatidylinositol 3-kinase (PI3K) Alpha iNhibition In Advanced Breast Cancer | [NCT02506556](https://clinicaltrials.gov/show/NCT02506556) | 2 | BYl719 |
|  | Study of Alpelisib (BYL719) in Combination With Trastuzumab and Pertuzumab as Maintenance Therapy in Patients With HER2-positive Advanced Breast Cancer With a PIK3CA Mutation | NCT04208178 | 3 | Alpelisib, Trastuzumab, Pertuzumab |
|  | Study Assessing the Efficacy and Safety of Alpelisib Plus Fulvestrant in Men and Postmenopausal Women with Advanced Breast Cancer Which Progressed on or After Aromatase Inhibitor Treatment (SOLAR-1) | NCT02437318 | 3 | Alpelisib, fulvestrant |
|  | Study Assessing the Efficacy and Safety of Alpelisib Plus Fulvestrant or Letrozole, Based on Prior Endocrine Therapy, in Patients With PIK3CA Mutation With Advanced Breast Cancer Who Have Progressed on or After Prior Treatments | NCT03056755 | 2 | Alpelisib, fulvestrant, letrozole, goserelin, leuprolide |
|  | Investigating Safety, Tolerability and Efficacy of AZD5363 When Combined With Paclitaxel in Breast Cancer Patients | NCT01625286 | 1/2 | AZD5363, paclitaxel |
|  | Testing the Addition of an Anti-cancer Drug, Copanlisib, to the Usual Maintenance Treatment (Trastuzumab and Pertuzumab) After Initial Chemotherapy in a Phase Ib/II Trial for Advanced HER2 Positive Breast Cancer | NCT04108858 | 1/2 | Copanlisib, Pertuzumab, Trastuzumab |
|  | Testing GDC-0032 (Taselisib) as a Potential Targeted Treatment in Cancers With PIK3CA Genetic Changes (MATCH-Subprotocol I) | NCT04439175 | 2 | Taselisib |
|  | Study of GDC-0941 or GDC-0980 With Fulvestrant Versus Fulvestrant in Advanced or Metastatic Breast Cancer in Participants Resistant to Aromatase Inhibitor Therapy | NCT01437566 | 2 | Fulvestrant, GDC-0941,  GDC-0980 |
|  | A Study of Prexasertib (LY2606368) With Chemotherapy or Targeted Agents in Participants With Advanced Cancer | NCT02124148 | 1 | Prexasertib |
|  | SAFIR PI3K A Phase II Randomized Trial Comparing Alpelisib and Fulvestrant Versus Chemotherapy as Maintenance Therapy in Patients With PIK3CA Mutated Advanced Breast Cancer | NCT03386162 | 2 | Alpelisib |
|  | A Study of BYL719 in Adult Patients With Advanced Solid Malignancies, Whose Tumors Have an Alteration of the PIK3CA Gene | NCT01219699 | 1 | BYL719, Fulvestrant |
|  | Open-label Phase 1b Study of ARQ 092 in Combination With Anastrozole | NCT02476955 | 1 | ARQ 092 |
| **AKT1/PIK3CA/mTOR** | Phase II Study of Herzuma® Plus Gedatolisib in Patients With HER-2 Positive Metastatic Breast Cancer | [NCT03698383](https://clinicaltrials.gov/show/NCT03698383) | 2 | Herzuma®, Gedatolisib |
|  | FUSCC Refractory TNBC Umbrella (FUTURE) | NCT03805399 | 1/2 | Pyrotinib, Capecitabine, mTOR inhibitor |
| **AKT1/PIK3CA/ESR1** | Study of the Molecular Features of Postmenopausal Women With HR+ HER2-negative aBC on First-line Treatment With Ribociclib and Letrozole and, in Patients With a PIK3CA Mutation, on Second-line Treatment With Alpelisib Plus Fulvestrant | NCT03439046 | 3 | Ribociclib, Letrozole, Alpelisib, Fulvestrant |
|  | Durvalumab, With Olaparib and Fulvestrant in Advanced ER+, HER2- Breast Cancer Patients. | [NCT04053322](https://clinicaltrials.gov/show/NCT04053322) | 2 | Durvalumab, Olaparib, Fulvestrant |
| **AKT1/PIK3CA/PTEN** | A Study of Ipatasertib in Combination With Paclitaxel as a Treatment for Participants With PIK3CA/AKT1/PTEN-Altered, Locally Advanced or Metastatic, Triple-Negative Breast Cancer or Hormone Receptor-Positive, HER2-Negative Breast Cancer | [NCT03337724](https://clinicaltrials.gov/show/NCT03337724) | 2/3 | Ipatasertib |
|  | Study Assessing the Efficacy and Safety of Alpelisib + Nab-paclitaxel in Subjects With Advanced TNBC Who Carry Either a PIK3CA Mutation or Have PTEN Loss Without PIK3CA Mutation | NCT04251533 | 3 | Alpelisib, nab-paclitaxel |
| **AKT1** | AZD5363 in Patients With Advanced Solid Tumors Harboring AKT Mutations | NCT03310541 | 1 | AZD5363, Enzalutamide, Fulvestrant |
|  | Safety, Tolerability & Potential Anti-cancer Activity of Increasing Doses of AZD5363 in Different Treatment Schedules | NCT01226316 | 1 | AZD5363 |
|  | Akt Inhibitor MK2206 in Treating Patients With Advanced Breast Cancer | NCT01277757 | 2 | MK2206 |
| **ESR1** | Evaluation of Lasofoxifene Versus Fulvestrant in Advanced or Metastatic ER+/HER2- Breast Cancer With an ESR1 Mutation | NCT03781063 | 2 | Lasofoxifene, Fulvestrant |
|  | PAlbociclib and Circulating Tumor DNA for ESR1 Mutation Detection | NCT03079011 | 3 | Palbociclib |
|  | Correlation of 16Î±-[18F]Fluoro-17Î²-estradiol PET Imaging With ESR1 Mutation | NCT03544762 | 3 | 18F-FES PET |
|  | Phase II Treatment of Metastatic Breast Cancer With Fulvestrant Plus Palbociclib or Tamoxifen Plus Palbociclib | NCT02913430 | 2 | Palbociclib, Fulvestrant, Tamoxifen |
|  | Evaluation of Lasofoxifene Combined With Abemaciclib in Advanced or Metastatic ER+/HER2− Breast Cancer With an ESR1 Mutation | [NCT04432454](https://clinicaltrials.gov/show/NCT04432454) | 2 | Lasofoxifene, Abemaciclib |
|  | The Efficacy of Fulvestrant in ESR1 (Estrogen Receptor 1) Mutated Metastatic Breast Cancer | [NCT03202862](https://clinicaltrials.gov/show/NCT03202862) | 2 | Fulvestrant |
| **ERBB2/HER2** | Neratinib HER Mutation Basket Study | [NCT01953927](https://clinicaltrials.gov/show/NCT01953927) | 2 | Neratinib |
|  | Neratinib +/- Fulvestrant in Metastatic HER2 Non-amplified But HER2 Mutant Breast Cancer | [NCT01670877](https://clinicaltrials.gov/show/NCT01670877) | 2 | Neratinib, Fulvestrant |
|  | A Study of Neratinib Plus Capecitabine Versus Lapatinib Plus Capecitabine in Patients With HER2+ Metastatic Breast Cancer Who Have Received Two or More Prior HER2 Directed Regimens in the Metastatic Setting (NALA) | [NCT01808573](https://clinicaltrials.gov/ct2/show/NCT01808573) | 3 | Neratinib, Lapatinib, capecitabine |
|  | A Study of Poziotinib in Patients With EGFR or HER2 Activating Mutations in Advanced Malignancies | NCT04172597 | 2 | Poziotinib Hydrochloride |
|  | A Phase II, Single-Arm Trial of Poziotinib as Salvage Treatment in Patients With Metastatic Breast Cancer Who Has HER2 or EGFR Mutation or Activated AR or EGFR Pathway | NCT02544997 | 2 | Drug: Poziotinib |
|  | A Study of BDTX-189, an Orally Available Allosteric ErbB Inhibitor, in Patients With Advanced Solid Tumors. | NCT04209465 | 1/2 | BDTX-189 |
| **TP53** | The p53 Breast Cancer Trial | NCT02965950 | 2 | Cyclophosphamide |
|  | Gene Therapy Plus Chemotherapy in Treating Patients With Breast Cancer | NCT00004038 | 1 | Ad5CMV-p53 |
|  | ALRN-6924 and Paclitaxel in Treating Patients With Advanced, Metastatic, or Unresectable Solid Tumors | NCT03725436 | 1 | ALRN-6924, Paclitaxel |
|  | Biomarker (p53 Gene) Analysis and Combination Chemotherapy Followed by Radiation Therapy and Surgery in Treating Women With Large Operable or Locally Advanced or Inflammatory Breast Cancer | NCT00017095 | 3 | Filgrastim, Chemotherapy, Radiation |
|  | Atezolizumab and Cobimetinib or Idasanutlin in Participants With Stage IV or Unresectable Recurrent Estrogen Receptor Positive Breast Cancer | NCT03566485 | 1/2 | Atezolizumab, Cobimetinib, Idasanutlin |
|  | Chemotherapy Combined With Gene Therapy in Treating Patients Who Have Stage III or Stage IV Breast Cancer | NCT00044993 | 2 | Ad5CMV-p53 gene, Chemotherapy |
|  | Safety and Efficacy of p53 Gene Therapy Combined With Immune Checkpoint Inhibitors in Solid Tumors. | NCT03544723 | 2 | Ad-p53 |
| **Mutations screening** | PErsonalized TREatment of High-risk MAmmary Cancer - the PETREMAC Trial (Predictive and prognostic value of mutations in 300 cancer-related genes assessed in breast cancer tissue by next generation sequencing before starting neoadjuvant therapy). | NCT02624973 | 2 | Tamoxifen,  goserelin,letrozole, palbociclib, docetaxel,olaparib, trastuzumab, pertuzumab, epirubicin |
|  | The UK Plasma Based Molecular Profiling of Advanced Breast Cancer to Inform Therapeutic CHoices (plasmaMATCH) Trial | NCT03182634 | 2 | Fulvestrant, Neratinib, AZD5363, Olaparib, AZD6738 |
|  | Targeted Therapy Directed by Genetic Testing in Treating Patients With Advanced Refractory Solid Tumors, Lymphomas, or Multiple Myeloma (The MATCH Screening Trial) | NCT02465060 | 2 | Adavosertib, Afatinib, Taselisib, Binimetinib, Capivasertib, Copanlisib, GSK2636771, Trametinib |
